# The phylogenetic and evolutionary analyses of detoxification gene families in *Aphidinae* species

**DOI:** 10.1371/journal.pone.0263462

**Published:** 2022-02-10

**Authors:** Rongmei Lin, Mengquan Yang, Bowen Yao

**Affiliations:** 1 Hubei Insect Resources Utilization and Sustainable Pest Management Key Laboratory, College of Plant Science & Technology, Huazhong Agricultural University, Wuhan, China; 2 CAS Key Laboratory of Insect Developmental and Evolutionary Biology, CAS Center for Excellence in Molecular Plant Sciences, Institute of Plant Physiology and Ecology, Chinese Academy of Sciences, Shanghai, China; 3 Graduate School of Pharmaceutical Sciences, The University of Tokyo, Bunkyo-ku, Tokyo, Japan; 4 School of Science, Beijing University of Chemical Technology, Chaoyang District, Beijing, China; Universita degli Studi della Basilicata, ITALY

## Abstract

Detoxification enzymes play significant roles in the interactions between insects and host plants, wherein detoxification-related genes make great contributions. As herbivorous pests, aphids reproduce rapidly due to parthenogenesis. They are good biological materials for studying the mechanisms that allow insect adaptation to host plants. Insect detoxification gene families are associated with insect adaptation to host plants. The *Aphidinae* is the largest subfamily in the *Aphididae* with at least 2483 species in 256 genera in 2 tribes: the *Macrosiphini* (with 3/4 of the species) and the *Aphidini*. Most aphid pests on crops and ornamental plants are *Aphidinae*. Members of the *Aphidinae* occur in nearly every region of the world. The body shape and colour vary significantly. To research the role that detoxification gene families played in the process of aphid adaptation to host evolution, we analyzed the phylogeny and evolution of these detoxification gene families in *Aphidinae*. In general, the P450/GST/CCE gene families contract, whereas the ABC/UGT families are conserved in *Aphidinae* species compared to these families in other herbivorous insects. Genus-specific expansions of P450 CYP4, and GST Delta have occurred in the genus *Acyrthosiphon*. In addition, the evolutionary rates of five detoxification gene families in the evolution process of *Aphidinae* are different. The comparison of five detoxification gene families among nine *Aphidinae* species and the estimated relative evolutionary rates provided herein support an understanding of the interaction between and the co-evolution of *Aphidinae* and plants.

## Introduction

Detoxification enzymes play important roles in anti-plant defense mechanisms when insects feed on plants because the insects metabolize deleterious compounds including insecticides and plant secondary metabolites [1] by using cytochrome P450 monooxygenases (P450s), carboxyl/ cholinesterases (CCEs), glutathione S-transferases (GSTs), UDP-glycosyltransferases (UGTs) and ATP-binding cassette transporters (ABCs) in their interactions with the host plants [2]. Furthermore, functional identification and verification of some genes have been performed via knocking out genes in five detoxification gene families [3, 4]. Moreover, the number of subfamilies and detoxification genes in different insect organisms varies widely [5, 6].

The insect detoxification enzyme system consists of a three-phase system, which is associated with biotransformation, metabolism, and excretion of toxic compounds [7]. Phase I detoxification enzymes include cytochrome P450 monooxygenase (P450s), esterase, and flavin monooxygenase, which can reduce the biological activity of a variety of endogenous toxic compounds and exogenous substances. Phase II enzymes mainly include glutathione transferases (GSTs) and UDP-glucuronyl transferases (UGTs), which act on toxic byproducts of phase I metabolism. Phase I and phase II detoxification enzymes are highly expressed in the midgut of insects, and phase III enzymes include ATP-binding cassette transporters (ABCs) and other transporters that export bound toxins to the extracellular level [7]. The structure, organization, and feature domains of five detoxification enzymes have been partially predicted by previous studies. In previous studies, some subfamilies showed associations with resistance to insecticides and secondary metabolites, playing important roles in anti-plant defense mechanisms; therefore, they can be defined as detoxification-related subfamilies, while other subfamilies were defined as detoxification-unrelated subfamilies. Research on detoxification-related subfamilies is essential because of their roles in the detoxification of harmful secondary metabolites of host plants.

Generally, five detoxification gene families detoxify deleterious compounds by different processes such as oxidation (P450s) and reduction (GSTs).

P450s are powerful biocatalysts, which produce nonactivated C–H bonds via catalyzing the introduction of one atom of molecular oxygen [8]. P450 can be divided into four clades: CYP2, CYP3, CYP4, and mitochondrial CYP [9]. Mitochondrial P450s play roles in metabolizing fatty acid, sterol, and hormones [9, 10]. CYP3 genes contribute to the oxidative detoxification of synthetic insecticides and plant secondary metabolites such as furanocoumarins, alkaloids [11, 12]. CYP4 genes are associated with detoxification and pheromone metabolism [12–14].

CCEs contribute to hydrolyzing pyrethroids and carbamates [15–17]. The CCEs fall into three main phylogenetic classes: the Intracellular catalytic class, the Secreted catalytic class, and the Neurodevelopmental class [13]. The Intracellular catalytic class, Beta esterase in the Secreted catalytic class, and acetylcholinesterase in the Neurodevelopmental class are the three parts associated with detoxification. For dietary/detoxification functions, CCE-A/B/C, Beta esterase, and CCEJ are related to the detoxification of harmful compounds [16].

GSTs conjugate deleterious compounds to the thiol group for more efficient degradation or excretion, which are involved in detoxifying insecticides, including spinosad, diazinon, nitenpyram, and DDT in insects [3, 18]. The Delta and Epsilon classes of the GSTs are unique to insects and are associated with the detoxification of insecticides. The Delta and Epsilon exist merely in insects and are involved in insecticide resistance [18]. Theta is believed to have given rise to cytosolic GSTs [19]. Microsomal GSTs, which are active as trimmers, are membrane-bound proteins. Cytosolic GSTs (primarily Delta and Epsilon gene members) are associated with resistance to DDT and organophosphates. Insects ordinarily have six different classes of GSTs [20].

In insects, UDP-glycosyltransferases (UGT) produce glycosides by catalyzing lipophilic compounds with sugars, playing important roles in the regulation of endobiotics and the detoxification of xenobiotics [5]. UGT signature motif, the N-terminal signal peptide and the C-terminal transmembrane domain are important domains [21]. Insect UGTs contribute to detoxification, sequestration, olfaction, and endobiotic modulation [22], which mainly exist in the fat body and midgut [23].

ABC genes are classified into eight subfamilies (A-H), of which the subfamilies A, B, C, and G are related to resistance to xenobiotics, including allelochemicals, multiple drugs, and insecticides [24]. ABCs hydrolyze ATP when transporting a wide variety of substrates across lipid membranes by two cytosolic nucleotide-binding domains (NBDs) and two transmembrane domains (TMDs) [25–28].

Aphids are important models of herbivorous adaptation. Aphids, which include approximately 5000 extant species, reproduce rapidly and indeed very damaging to crop plants predominantly because of parthenogenesis and sap-sucking, not only reducing plant production but also spreading a wide variety of plant viruses. Some aphids exhibit several kinds of life cycle including periodic parthenogenesis, meaning asexual alternates with sexual reproduction respond to seasonal changes, during which aphids shift to a new host plant belonging to another family [29], while pea aphids stay on the same host plant during sexual reproduction. Aphids can be divided into oligophagous and polyphagous aphids by their eating habits [29]. Aphids present different phenotypes in different host plants. The size of the aphid, the growth rate, and the reproduction rate vary widely when feeding on different host plants, especially for polyphagous aphids [29]. The host plants, feeding parts, and defense mechanisms against plants of aphids are diverse. Some aphids evolved multiple biotypes that exhibit host adaption differences such as *A*. *gossypii* [30]. The *Aphidinae* is the largest subfamily with at least 2483 species in 256 genera in 2 tribes: the *Macrosiphini* (with 3/4 of the species) and the *Aphidini*. Most *Aphidinae* live on angiosperms, but a small number of secondarily adapted species attack conifers and ferns. Some or all species in many genera undergo a regular seasonal host alternation (heteroecy) between a woody perennial primary host, on which the diapausing eggs overwinter, and one or more herbaceous secondary hosts, to which some or all of the populations migrate by means of alate viviparae during the spring and early summer. Most aphid pests on crops and ornamental plants are *Aphidinae*. Members of the *Aphidinae* occur in nearly every region of the world. The body shape and color vary significantly.

In previous studies about detoxification gene families, some research has focused on one or two insect species and has analyzed some of these five detoxification gene families, while other studies compare one detoxification gene family among several insect species. For example, Rispe et al. research the phylogenetic of the five classes for phylloxera and two aphid species. The evolutionary and phylogenetic circumstances of detoxification-related subfamilies are especially unknown. *Aphidinae* is the most diverse major lineage of aphids (*Aphididae*), which dominate the temperate, northern-hemisphere fauna. Therefore, we performed systematic phylogenetic and evolutionary analysis on five detoxification gene families to study the phylogenetic and global evolutionary circumstances of detoxification-related genes in *Aphidinae*.

Nine common and harmful aphid species were studied in this study; these species include five oligophagous *Aphidinae* and four polyphagous *Aphidinae*.

## Materials and methods

### Gene annotations

The protein databases of the insect species were downloaded from AphidBase, Whitefly Genome Database, FlyBase, and NCBI (https://www.ncbi.nlm.nih.gov). All collected ABC gene sequences of *A*. *pisum* and *D*. *melanogaster* were searched against another aphid species’ protein database by applying BLASTP with an e-value threshold of 1e-5. Gene sequences of *A*. *pisum* were searched against another aphid species’ protein database for the other four gene families. All collected gene sequences of *A*. *pisum* were searched against the other eight species’ protein databases by applying BLASTP for the five gene families. In turn, the above databases were exchanged with seed and BLASTed a second time. InterProScan [31] was used to screen all predicted protein sequences and filter the ones without characteristic domains [32]. Each gene of five gene families was annotated manually.

### Insects phylogenetic analysis

We fitted the phylogenetic structure of seventeen insect species by synthesizing the relative phylogenetic relationships obtained by each species taken from references [33, 34].

We reconstructed the phylogenetic topology of nine *Aphidinae* species and eight other herbivorous insects, including *Daktulosphaira vitifoliae* [35], *B*. *tabaci* [36], *N*. *lugens* [37], *Tribolium castaneum* [38], *L*. *decemlineata* [39], *S*. *litura* [24], *P*. *xylostella* [40], and *D*. *plexippus* [41]. The phylogenetic topology of the nine *Aphidinae* species was taken from Carol et al. [33]. The phylogenetic topology of the other eight species was taken from Behura. et al [34]. The tree was rerooted using five insects from Lepidoptera and Coleoptera.

We applied MUSCLE to perform multiple alignments and inspect manually for well aligned blocks. Poorly aligned regions of multiple alignments were discoverd and discarded before the phylogeny analysis. We used PhyML with the JTT model and 1000 bootstrap replicates to implement the phylogenetic analysis. Subfamilies of each family were divided according to *D*. *melanogaster* (ATP-binding cassette transporter gene family) or *A*. *pisum* (four other gene families) genes.

### Estimation of gene gain and loss events

The parsimony-based ‘modified reconciliation method’ was used to estimate the number of gene gain and loss events; First, branches with bootstrap support above 70 in phylogenies of detoxification gene families were screened out. Then, the Delta/ CYP4 phylogenetic tree and nine *Aphidinae* species phylogenetic tree were uploaded to Notung [42], which reconciled the condensed trees with the organismal relationships.

### Amino acid sequence identity

Local BLAST was used to calculate the amino acid sequence identity within or among *Aphidinae* species. For each group (x-axis), every amino acid sequence (n) of each *Aphidinae* species was blasted against each other and produced nx(n-1)/2 identity numbers for each *Aphidinae* species. The average of the identity is indicated in the colored hollow circle for each *Aphidinae* species (Fig 4A). For each group (x-axis), all amino acid sequences of the nine *Aphidinae* species blasted against each other and produced a file with twelve columns; then, the rows that repeated with the previous nine files within the *Aphidinae* species from Fig 4A were discarded; subsequently, the remaining identities were extracted, producing not only an average and standard deviation (Fig 4B), but a boxplot as well (Fig 4C).

## Results

### Species selection

In subfamily *Aphidinae*, *A*. *kondoi*, *A*. *pisum*, *M*. *persicae*, *D*. *noxia*, *S*. *avenae* belong to tribe *Macrosiphini*, the other species belong to tribe *Aphidini*. Some are host-alternating species, such as *M*. *persicae* [43], *Rhopalosiphum padi* [44], and *Aphis glycines* [45]. *A*. *pisum* is the first aphid species that had its genome completely sequenced [46, 47]. *Acyrthosiphon kondoi* does more damage to leguminous plants than *A*. *pisum* does, severely diminishing the crop yields even at low population densities, especially in spring and autumn [48]. *Aphis gossypii* is widely distributed in diverse crops in the families Malvaceae, Rutaceae, and Cucurbitaceae [29]. *Sitobion avenae* [49], *Diuraphis noxia* [19], and *Schizaphis graminum* [50] are injurious insects that feed on Gramineae family members. Furthermore, *A*. *pisum*, *A*. *kondoi*, *A*. *glycines*, *D*. *noxia*, and *S*. *graminum* are oligophagous, and *A*. *gossypii*, *M*. *persicae*, *R*. *padi*, and *S*. *avenae* are polyphagous.

### Comparison of detoxification-related genes and phylogenetic topology of seventeen insect species

**Fig 1:**
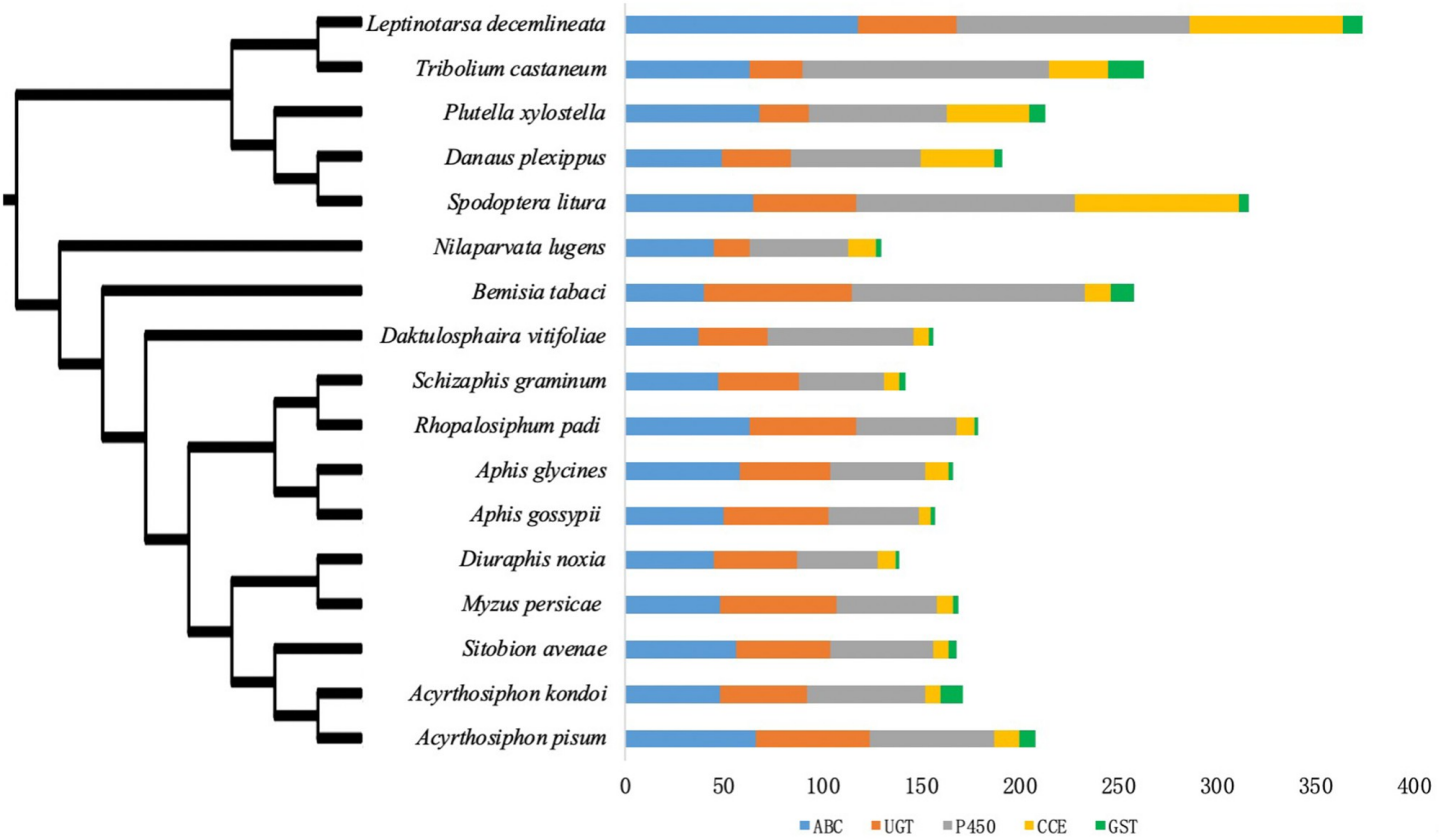
Phylogenetic topology and detoxification gene numbers of seventeen insect organisms. The phylogenetic topology of nine *Aphidinae* species and eight other phytophagous insect organisms was taken from papers. The tree was rooted using five insect organisms from the *Lepidoptera* and *Coleoptera*. Gene counts are the total numbers of the ATP-binding cassette transporter, cytochrome P450 monooxygenases, glutathione S-transferases, carboxylesterase and UDP-glycosyltransferases.

To compare the detoxification-related genes among *Aphidinae* and other herbivorous insects, genes from five detoxification gene families were identified from 17 insect protein databases and were divided into subfamilies, whereupon genes from the detoxification-associated subfamilies became the focus. In general, the P450/GST/CCE genes contract, whereas the ABC/UGT genes are conserved in *Aphidinae* compared with other herbivores (S1–S6 Figs, S1 Table). Total numbers of detoxification-associated genes of P450 varied from 41 to 63 in nine *Aphidinae* species, whereas they varied from 50 to 125 in the other eight insect organisms; furthermore, more than 110 detoxification-related P450 genes were observed in four insect species, which indicates that the detoxification-associated P450 genes contract in *Aphidinae* compared with other phytophagous insects. Total counts of detoxification-related genes of CCE vary from 6 to 13 in nine *Aphidinae* species, whereas they vary from 13 to 83 in the other eight insect organisms; furthermore, more than 30 detoxification-associated CCE genes were observed in five insect species, which suggests that detoxification-related CCE genes contract in *Aphidinae* compared with other herbivorous insects. GST detoxification-associated genes vary from 2 to 11 in nine *Aphidinae* species, whereas they vary from 2 to 18 in the other eight insect organisms; furthermore, no more than 4 detoxification-associated GST genes were observed in seven aphid species, and more than 8 detoxification-associated GST in other eight insect organisms, which indicates that detoxification-related GST genes contract in *Aphidinae* compared with other herbivorous insects. The average UGT gene count is 49 in nine *Aphidinae* species and 35 in the remaining seven insect organisms except for *B*. *tabaci*, which suggests that, generally, UGTs expand in *Aphidinae* compared with other herbivorous insects. The number of detoxification-associated genes of ABC varies from 40 to 68 in fifteen insect organisms, except for *Leptinotarsa decemlineata* and *Daktulosphaira vitifoliae* [34], indicating that detoxification-related ABCs are conserved in *Aphidinae* compared with other herbivorous insects. Generally, P450/GST/CCE detoxification-related genes contract, whereas ABC/UGT are conserved in *Aphidinae* compared with other herbivorous insects (Fig 1, S6 Fig).

### Some detoxification-unrelated subfamilies in GST/CCE/P450 are conserved in *Aphidinae*

**Fig 2:**
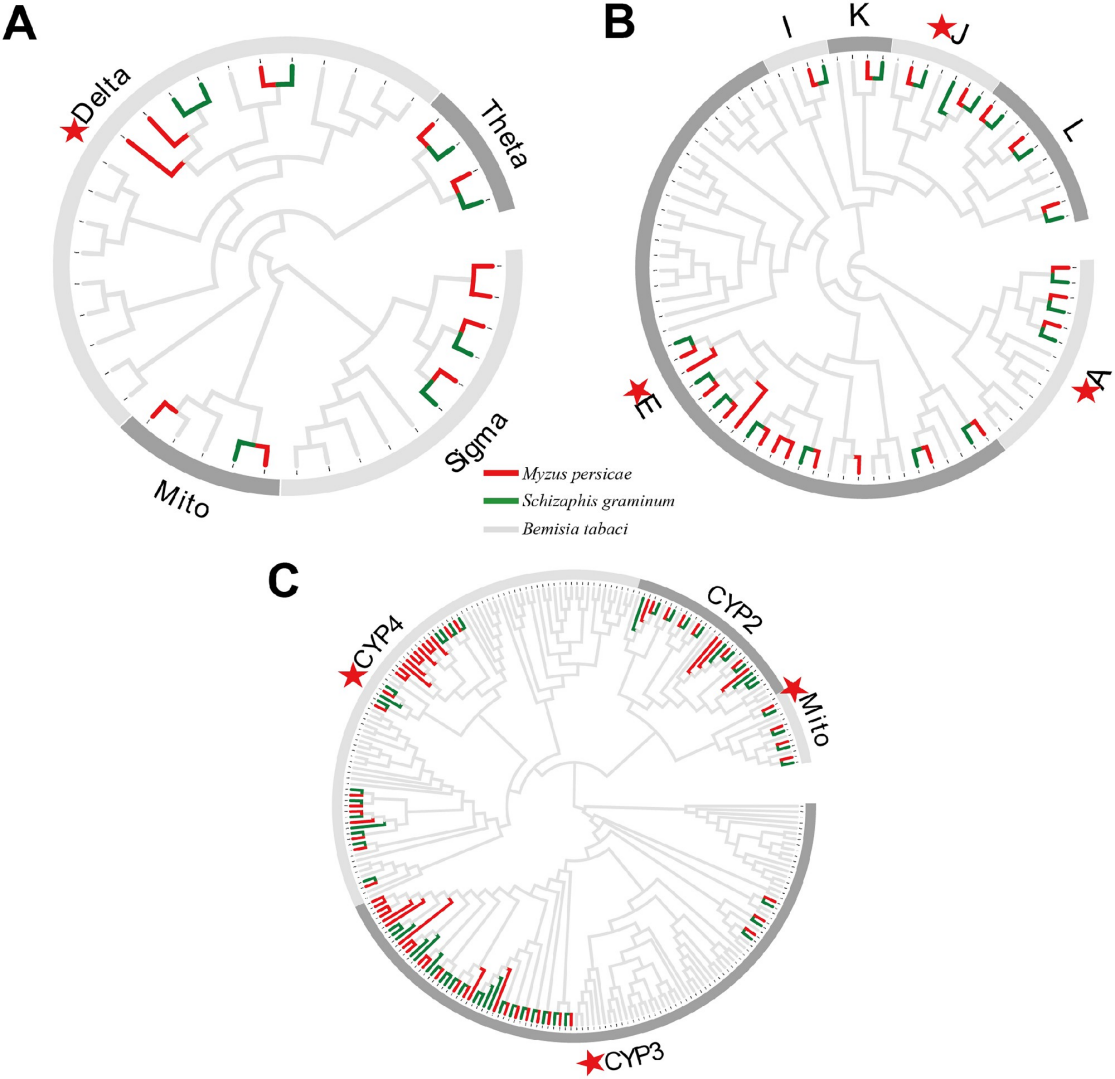
Analysis of three gene families (GST, CCE, P450) in two *Aphidinae* species and *Bemisia tabaci*. These two *Aphidinae* species are from two different *Aphidinae* branches of the phylogenetic topology shown in Fig 1. *M*. *persicae* belong to tribe *Macrosiphini*, *S*. *graminum* belong to tribe *Aphidini*. These three gene families contract in *Aphidinae* compared with other insect organisms. (A) GST, (B) CCE, (C) P450. Genes in different species are color-coded as follows: *Myzus persicae*, red; *Schizaphis graminum*, green; *B*. *tabaci*, grey. Red stars indicate detoxification-related subfamilies.

Within nine *Aphidinae* species, according to our division of subfamilies in the five gene families (S1–S5 Figs, Fig 2), the considerably different patterns of expansion/contraction displayed by the subfamilies of these three gene families can be divided into three types: 1) Only a single-copy gene of the K subfamily occurs in each *Aphidinae* species, and only a single-copy gene of the I subfamily occurs in each *Aphidinae* species except for *S*. *avenae* and *A*. *glycines*, which have two copies; 2) Theta, Microsomal GST, Neuro/developmental class (K, L, J, I) of CCE, CYP2, Mitochondrial of P450, and A/B/D/E/F of ABC lost more genes than were duplicated; and 3) Delta, Sigma of GST, A, E of CCE, CYP3, CYP4 of P450 and C/G/H of ABC had expanded in *Aphidinae* species.

Among *Aphidinae* and other herbivorous insects, detoxification-related subfamilies contracted compared with those observed in other insects, whereas some of the detoxification-unrelated subfamilies were conserved in *Aphidinae*. For instance, in the GST Delta subfamily, three *M*. *persicae* genes and three *S*. *graminum* genes were observed, whereas 14 *B*. *tabaci* genes (Fig 2A) were observed. However, CCEJ is conserved in that CCEJ belongs to the Neuro/ developmental class (Fig 2B). Whereas two cases of detoxification-unrelated subfamilies were present in *Aphidinae*, some subfamilies, such as sigma, contracted, and in some subfamilies, such as CYP2 (Fig 2C), *Aphidinae* conserved. Although the whole gene family contracts, some of the detoxification-unrelated subfamilies were conserved in *Aphidinae*. CYP3 and CYP4 subfamilies incorporate 77.2% of the entire repertoires of the P450 genes in the nine *Aphidinae* species. *Aphidinae* species have a decreased number of P450 genes compared with the number observed in other insects. *Aphidinae* occupies six subfamilies of the CCE, namely, A/E/I/J/K/L. This E subfamily incorporates 44.7% of the entire repertoires of the CCE genes in the nine *Aphidinae* species. However, *R*. *padi* esterase genes have undergone an expansion within clade E compared with the other eight *Aphidinae* (S2 Fig). A large fraction of the CCE proteins is associated with basal metabolic functions that are presumably the same or similar in *Aphidinae*.

### Genus-specific expansion of CYP4 and Delta occurred in *Acyrthosiphon*

**Fig 3:**
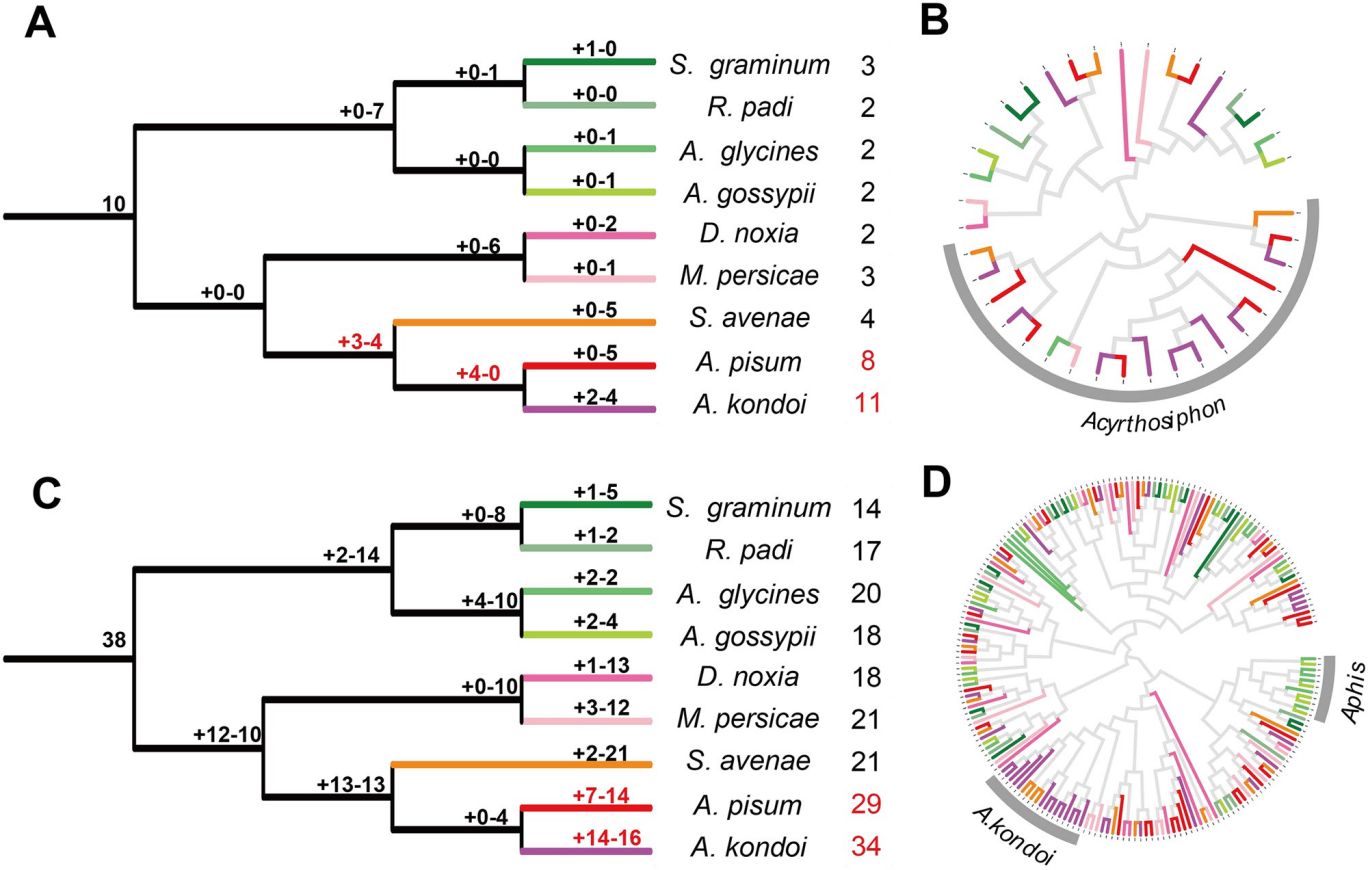
Phylogenetic relationships and gene gain-and-loss events of two subclades. Genus- specific expansions of CYP4 and Delta occur in the genus *Acyrthosiphon*. Notung was used to calculate numbers of gene gain-and-loss, and numbers are presented above branches with negative and plus signs, respectively. Maximum-likelihood trees of the CYP4 and Delta genes were developed by using PhyML. (A) Gene gain-and-loss events of Delta subfamily. Most duplications occurred before formation of the genus *Acyrthosiphon* and are indicated in red. (B) The phylogenetic tree of the Delta subfamily in nine *Aphidinae* species. Clade for most of the genus *Acyrthosiphon* is highlighted in grey arc. (C) Gene gain-and-loss events of CYP4 subclade. Most duplications occurred after formation of the genus *Acyrthosiphon* and are indicated in red. (D) Phylogenetic tree of CYP4 clade in nine *Aphidinae* species. The clade for *A*. *kondoi* most and the clade for the genus *Aphis* only are highlighted in the grey arc, respectively. Red palette indicates the tribe *Macrosiphini*, green palette indicates the tribe *Aphidini*. Genes in different species are colour-coded as follows: *S*. *graminum*, green; *R*. *padi*, dark sea green; *A*. *glycines*, spring green; *A*. *gossypii*, green yellow; *D*. *noxia*, hot pink; *M*. *persicae*, pink; *S*. *avenae*, dark orange; *A*. *pisum*, red; and *A*. *kondoi*, magenta. The numbers after the aphid species names indicate numbers of Delta or CYP4 genes in the extant aphid species.

According to phylogenetic analysis, eleven detoxification-related subfamilies in these gene families were divided, *Aphidinae*’ genes from each detoxification-related subfamily were screened to infer the phylogenetic tree of each subfamily (Fig 3, S6 Fig), wherein genus-specific expansion in *Acyrthosiphon* occurred in two subfamilies: CYP4 and Delta (Fig 3B and 3D). In most of these eleven subfamilies, gene counts of *D*. *noxia* were low, whereas those of *A*. *pisum* were high. The genus *Aphis* expanded in two clades. The branch for *A*. *glycines* in CYP3 contained five CYP9E2 genes. The clade for the genus *Aphis* comprises *A*. *glycines* and *A*. *gossypii* and contains eleven CYP380C6 genes only, which contribute to the spirotetramat resistance of *A*. *gossypii* (Fig 3D). CYP380C6 genes contribute to spirotetramat resistance at very high resistance levels.

We appraised the gene gain and loss events and assessed the number of ancestral gene copies in each subfamily to further deduce the evolutionary dynamics of detoxification-related genes. *A*. *pisum* and *A*. *kondoi* genes distributed uniformly, mostly among the branch for the genus *Acyrthosiphon* (Fig 3B). The clade for *A*. *kondoi* mostly does not contain *A*. *pisum* genes, which differs from the Delta subfamily. Delta genes in the genus *Acyrthosiphon* are nearly 3.7 times more common than in the other seven aphid species. The numbers of Delta genes in the other seven aphid species decrease to a quarter of those present in the ancestor of the *Aphidinae*.

According to the parsimony analysis, the number of Delta and CYP4 genes in the genus *Acyrthosiphon* likely remained the same as the common ancestor while decreasing greatly in other *Aphidinae*. Considerable gene losses occurred in the *Aphidinae*. In general, *Aphidinae* lost more Delta and CYP4 genes than were duplicated in the whole process of evolution, the GST-Delta and CYP4 genes have distinct evolutionary dynamics in *Acyrthosiphon* in comparison to other *Aphidinae*.

Many more gene-gain events occurred with Delta either before the separation of the genus *Acyrthosiphon* from *S*. *avenae* or before the formation of the genus *Acyrthosiphon* (Fig 3A), whereas many more gene-gain events occurred with CYP4 either before the separation of the genus *Acyrthosiphon* from *S*. *avenae* or after the formation of the genus *Acyrthosiphon* (Fig 3C). Duplications mainly occurred in the different stages in these two sub-clades. Most duplications of the *Acyrthosiphon* CYP4 genes occurred during the later stages of *Acyrthosiphon* evolution than the GST-Delta genes.

### Comparison of relative evolutionary rates among gene families and subfamilies

**Fig 4:**
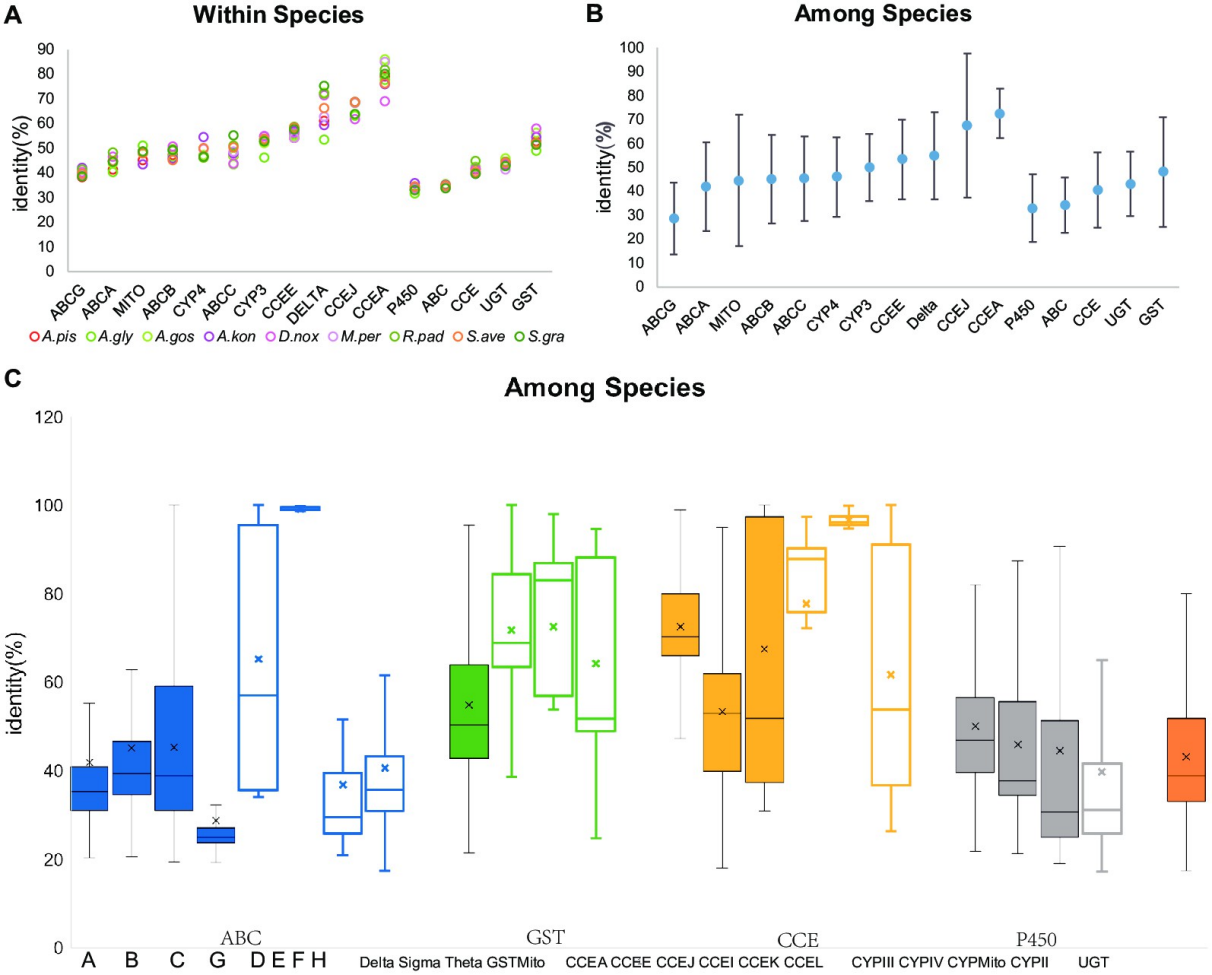
Relative evolutionary rate reflected by amino acid sequence identity. The upper line, middle line, lower line and error symbol indicate upper quartile, median, lower quartile and average of each box, respectively. (A) The amino acid sequence identity within nine *Aphidinae* species of eleven detoxification-related subfamilies and five detoxification families is exhibited by scatter diagram. Colors of species are the same as in Fig 3. (B) Sequence identity among nine *Aphidinae* species of the eleven detoxification-related subfamilies and five detoxification families is displayed by scatter diagram. (C) Sequence identity among nine *Aphidinae* species of eleven detoxification-related subfamilies, eleven detoxification-unrelated subfamilies and the UGT family is exhibited by box plot. Detoxification-related subfamilies are displayed in solid boxes, whereas the detoxification-unrelated subfamilies are hollow boxes. Colors of detoxification families are the same as in Fig 1.

The rank of the evolutionary rate within or among *Aphidinae* species for both detoxification gene families and subfamilies are similar. The rank of the evolutionary rates within or among *Aphidinae* species (from fast to slow) for total subfamilies is ABC P450 CCE GST and for total gene families is P450 ABC CCE UGT GST. The higher the sequence identity, the lower the evolutionary rate. ABCG evolves the fastest, whereas CCEA evolves the slowest within or among the *Aphidinae* species. In general, ABC evolves the fastest, whereas GST evolves the slowest within (Fig 4A) or among (Fig 4B) the *Aphidinae* species for the total subfamilies. However, P450 evolves the fastest, whereas the GST evolves the slowest within (Fig 4A) or among (Fig 4B) the *Aphidinae* species for the total gene families, and P450 evolves faster than ABC for the total gene families. These results suggest that, in general, the rankings of the evolutionary rates within *Aphidinae* species are the same as among the *Aphidinae* species; nothing but CYP4 changes the order with ABCC, which does not affect the rank of the total subfamilies. Detoxification-related subfamilies of P450 are more conserved than detoxification-unrelated subfamilies among *Aphidinae* species, which is contrary to observations made of ABC/GST/CCE (Fig 4C). The P450 evolves the fastest among the five gene families, not only within species but also among the species, whereas the detoxification-related subfamilies evolve more slowly than the detoxification-unrelated subfamilies. Detoxification-related subfamilies of P450 are more conserved than detoxification-unrelated subfamilies (CYP2) among *Aphidinae* species. In addition, GST is the most conserved among the five gene families, not only within but also among species (Fig 4A and 4B). Furthermore, the detoxification-unrelated subfamilies of ABC/GST/CCE are more conserved than detoxification-related subfamilies among *Aphidinae* species (Fig 4C), which indicates that, to better adapt to host plants and the changing environment, detoxification-related genes of the ABC/GST/CCE gene families evolve faster and generate diverse genes. However, as for P450, each of these three detoxification-related clades comprises a wide variety of detoxification-related subfamilies, which encompass diverse detoxification-related genes. Although the number of detoxification-related genes is not very large, the variety of genes is enough.

Generally, the evolutionary rates of five detoxification gene families in the evolution process of *Aphidinae* are different.

## Discussion

Duplications of detoxification-related genes in *Aphidinae* are not obvious, and consequently, the detoxification-related gene numbers are lower. In a previous study, they concluded that P450 is consistent with the hypothesis that the broader the host range is, the more detoxification genes the species owns. GST and CCE aren’t consistent with the hypothesis, which is conformed to our results [1]. Without the genome of *M*. *persicae*, they used *M*. *persicae* cDNA while we used the protein database. The method of searching candidate genes in their study is different from ours.

The host range of oligophagous aphid species (*A*. *glycines*, *A*. *kondoi*, *A*. *pisum*, *D*. *noxia*, and *S*. *graminum*) is narrow, whereas polyphagous aphid species (the other four aphid species) feed on hundreds of species in a wide variety of plant families. In subfamily *Aphidinae*, *A*. *kondoi*, *A*. *pisum*, *M*. *persicae*, *D*. *noxia*, *S*. *avenae* belong to tribe *Macrosiphini*, the other species belong to the tribe *Aphidini*. Accordingly, polyphagous species would be exposed to a higher diversity of insecticides and plant secondary metabolites than oligophagous species and have been predicted to require a greater complement of detoxification-related enzymes [1, 43, 51]. Polyphagous species have more detoxification genes than oligophagous species for Lepidoptera; for instance, *S*. *litura possesses* the most detoxification genes, and *D*. *plexippus* possess the least among the three Lepidoptera species; these occurrences correspond to the host ranges of the three species. The Hemipterans *B*. *tabaci* and *N*. *lugens* adapt to this rule, and the polyphagous *B*. *tabaci* possess more detoxification genes than the oligophagous *N*. *lugens*. However, the number of detoxification-associated genes appears irrelevant to the host ranges in *Aphidinae* species. Species that are named based on host plants have narrower host ranges. The names of *Aphidinae* species are usually based on the host plant names regardless of whether the aphid species are polyphagous or oligophagous. Our research implies that *Aphidinae* does not require different genes to detoxify diverse plant secondary metabolites and insecticides, as one specific detoxification-related gene may be able to detoxify several different toxic substances [1, 43].

Three contracted gene families suggest that the significance of P450/GST/CCE to *Aphidinae* is smaller than the significance of P450/GST/CCE to other herbivorous insects. *Aphidinae* possesses much smaller repertoires of these three detoxification gene families than other herbivorous insects, likely indicative of a simpler detoxification system. Genes with conserved roles tend to occupy relatively stable copy numbers while those with diversified functions have higher rates of gain-and-loss, although the degrees of copy number changes are somewhat random. Our results indicate that this pattern could also hold true for the evolution of detoxification gene families of *Aphidinae*. For instance, CCE-K presents one single-copy in each of the nine *Aphidinae* species, which suggests that CCE-K is possible to perform a similar function common for all *Aphidinae*.

Two conserved gene families suggest that the significance of ABC/UGT to *Aphidinae* is the same as the significance of ABC/UGT to other herbivorous insects. Except for *B*. *tabaci*, generally, UGTs expand in *Aphidinae* compared with other herbivorous insects [36].

Detoxification-unrelated subfamilies of ABC/GST/CCE are more conserved than detoxification-related subfamilies among *Aphidinae* species, which indicates that the detoxification-related genes of these three gene families evolve faster and generate diverse genes to adapt to changing environments. However, as for P450, CYP2 is the only detoxification-unrelated subfamily in P450 [52]. Each of these three detoxification-related clades comprises a wide variety of detoxification-related subfamilies, which encompass diverse detoxification-related genes. The proportion of detoxification-related genes in P450 is much higher than the proportion of detoxification-related genes in ABC/GST/CCE. Thus, detoxification-related P450s are more plentiful for *Aphidinae* compared with ABC/GST/CCE. We predicted that these might be potential reasons for the detoxification-related clades of P450 being more conserved than the detoxification-unrelated clades among *Aphidinae* species, which is contrary to ABC/GST/CCE (Fig 4C).

Genus-specific expansions of CYP4 and Delta have occurred in the genus *Acyrthosiphon*, and these expansions happened mainly before the formation of the genus *Acyrthosiphon* in Delta and predominantly after the formation of *Acyrthosiphon* in CYP4, which reflect differences in their feeding habits and detoxification situation. The number of Delta and CYP4 genes in the genus *Acyrthosiphon* probably remained the same as that of the common ancestor, whereas it greatly decreased in other *Aphidinae*. Possibly, dramatic decreases in gene numbers in the other extant seven *Aphidinae* species are caused by *Aphidinae* losing genes with redundant and unnecessary functions in the evolutionary process and may help explain why different genus feed on different host plants [53].

Delta genes in the genus *Acyrthosiphon* are nearly 3.7 times as common as those observed in the other seven *Aphidinae* species. This occurrence suggests that the genus *Acyrthosiphon* needs more Delta genes to detoxify both the xenobiotic and endogenous harmful compounds (S3 Fig). Delta and Epsilon comprise approximately half of the GSTs present in an insect and are involved in insecticide resistance. However, generally, *Aphidinae* lacks the Epsilon, Omega, and Zeta classes compared to the other herbivorous insects [1]. Insects ordinarily occupy six different subfamilies of GSTs [18, 54]. The activation of the mitogen-activated protein kinase (MAPK) pathway was mediated by Omega GSTs [55]. Zeta and Omega GSTs protect insects against oxidative stress [55, 56]. Epsilon class genes are related to resistance to the organochlorine insecticide DDT [1,1,1-trichloro-2,2-bis-(p-chlorophenyl) ethane] [57]. Sigma GSTs contribute to lipid peroxidation and detoxification. Delta GSTs are also associated with resistance to organophosphate/DDT, so *Aphidinae* may not need the Epsilon subfamily. Because of the high proportion of Delta serving the same function as Epsilon, *Aphidinae* lost Epsilon in the process of evolution. The *Aphidinae* may also have lost Zeta and Omega subfamilies since microsomal GSTs contribute to protecting insects against oxidative stress and provide the function normally provided by the Zeta and Omega GSTs.

As for CCE, in general, *Aphidinae* lack the unique CCE subfamilies of other insect orders such as the Diptera-specific clades B and C, dipteran juvenile hormone esterases (F), lepidopteran juvenile esterases (G), and six of fourteen subfamilies are represented in *Aphidinae*. These subfamilies include intracellular catalytic class A, which is involved in dietary/detoxification; secreted catalytic class E, which is related to hormone/semiochemical processing and contains juvenile hormone esterase and Beta esterase; neurodevelopmental classes I/J/K/L (I, Glutactin; K, Gliotactins; J, Acetylcholinesterase; L, Neuroligins) [1], which means *Aphidinae* lack eight subfamilies compared with other herbivores; class M (the glutactin like esterases (H), lepidopteran juvenile esterases (G), dipteran juvenile hormone esterases (F), integument esterases (D)); and the Diptera-specific clades B/C, and are all clades without detectable aphid homologues. A decrement in the diversity of CCEs associated with hormone and pheromone processing (clades D-H) with only clade E having any gene members at all was observed in the *Aphidinae* species [58]. Gene members of the clades I–M (Neurodevelopmental class) tend to be noncatalytic and are relevant to cell-cell interactions, except for acetylcholine esterase (Ache, clade J) [59].

The genus *Aphis* expands into two clades of detoxification-related subfamilies. Branch for *A*. *glycines* only in CYP3 comprises five CYP9E2 genes. CYP9E2 genes contribute to imidacloprid detoxification [60]. The clade for the genus *Aphis* comprises *A*. *glycines* and *A*. *gossypii* and contains eleven CYP380C6 genes, which contribute to the spirotetramat resistance of *A*. *gossypii* (Fig 3D). The CYP380C6 genes contribute to spirotetramat resistance at very high resistance levels. Possibly, CYP9E2 and CYP380C6 duplicate to enhance the efficiency of detoxifying the common insecticides to which *Aphidinae* are exposed.

As for P450, all insects had four clades, with each clade containing several subfamilies. We just divided P450 into four clades, whereas the P450 did not subdivide into subfamilies in the *Aphidinae*. Although genes exist in each of the four clades, we were unable to determine if genes in every subfamily exist in every clade. In this study, we generally refer to the four clades of P450 as four subfamilies, and possibly, the *Aphidinae* lack several subfamilies in P450. Therefore, GST/CCE/P450 is inferred to contract in *Aphidinae* because they lack several subfamilies compared with other herbivorous insects.

Expanded subfamilies contract in contracted detoxification gene families, such as P450/GST/CCE, whereas the expanded subfamilies expanded in contrast to the detoxification gene families, such as ABC, in *Aphidinae*. Our study implies that for commonly expanded subfamilies, the evolutionary dynamics are generally consistent with the global gene family, whereas, for commonly contracted subfamilies, such as CYP2, the evolutionary dynamics are sometimes opposite to the global gene family. That is, CYP2 contracts in all insects, whereas it expands in *Aphidinae* compared with other herbivorous insects (Fig 2C).

For the approach of comparison of relative evolutionary rates, taking blast hits amino acid identity could be acceptable, since the comparison is comprehensive (different levels: within or among *Aphidinae* species, *Aphidinae* with other herbivorous insects, and among gene families and subfamilies) and reflect each sequence. For each group (x-axis), every amino acid sequence (n) of each *Aphidinae* species was blasted against each other and produced nx(n-1)/2 identity numbers for each *Aphidinae* species. For each group (x-axis), all amino acid sequences of the nine *Aphidinae* species blasted against each other and produced a file with twelve columns; then, the rows that repeated with the previous nine files within the *Aphidinae* species were discarded; subsequently, the remaining identities were extracted, producing an average and standard deviation. However, there exists some possibly important caveats. Indeed, for some relatively divergent gene families, the blast analysis might give different results for the same gene, with quite different identities. Therefore, it might be difficult to select the most relevant metric of genetic distance, which is a potential limitation. Of course, there might be problems also if using full-length alignments that might be cleaned in divergent regions. Ks value reflect comparative evolutionary rates between two species during the same time period. Here, we compare nine species so we use identity to compare. Essential genes are known to evolve slowly. The rank of the evolutionary rates within or among *Aphidinae* species (from fast to slow) for total subfamilies is ABC P450 CCE GST and for total gene families is P450 ABC CCE UGT GST, which indicates that phase II detoxification enzymes (GST, UGT) are more essential than other detoxification enzymes. The rank of the evolutionary rates is the same within or between *Aphidinae* species, which suggests that amino-acid sequences are similar in each detoxification gene family within *Aphidinae*. Therefore, for each phylogenetic tree of detoxification gene family, genes of each subfamily from nine *Aphidinae* species cluster together.

## Conclusion

In this study, we identified the genes of five detoxification gene families in seventeen insect organisms (nine *Aphidinae* species and eight other herbivorous insects); calculated the phylogenetic relationships; divided the five gene families into several subfamilies, which comprise detoxification-related and detoxification-unrelated subfamilies; and estimated the relative evolutionary rates among the gene families and subfamilies.

In general, the P450/GST/CCE gene families have contracted, whereas the ABC/UGT are conserved in *Aphidinae* compared with other herbivorous insects, and the numbers of detoxification-associated genes appear irrelevant to the host ranges of the *Aphidinae* species. P450/GST/CCE detoxification-associated genes have contracted in *Aphidinae* compared with other herbivorous insects, whereas some detoxification-unrelated subfamilies are conserved in *Aphidinae*.

Furthermore, the genus-specific expansion of P450, CYP4, and GST Delta have occurred in the genus *Acyrthosiphon*, and this happened in Delta either before the separation of the genus *Acyrthosiphon* from *S*. *avenae* or before the formation of the genus *Acyrthosiphon*, and in CYP4 either before the separation of the genus *Acyrthosiphon* from *S*. *avenae* or after the formation of the genus *Acyrthosiphon*. Moreover, the evolutionary rates of the five detoxification gene families in the evolutionary process of *Aphidinae* are different, and the rankings of the evolutionary rate within or among aphid species for both detoxification gene families and subfamilies are similar. In ABC/GST/CCE, detoxification-related genes evolved faster than detoxification-unrelated genes; in contrast, detoxification-related clades of P450 are more conserved than detoxification-unrelated clades among *Aphidinae* species.

Our study is important in the field of detoxification gene families in *Aphidinae*, including the comparisons at several different levels (within or among *Aphidinae* species, *Aphidinae* with other herbivorous insects, and among gene families and subfamilies). The identification of these detoxification genes, the comparison of five detoxification gene families between nine *Aphidinae* species, and the relative evolutionary rates we estimated provide understanding of their contribution to the adaptation of *Aphidinae*. Our study serves as the basis for analyzing the evolutionary circumstances of detoxification genes in insects and the important roles that detoxification enzymes play in the interaction between insects and host plants. Moreover, we provide support for analyzing co-evolution between *Aphidinae* and plants and ecological interaction.

## Supporting information

S1 Fig Phylogenetic tree of ABC in *Aphidinae*. ABC is divided into eight subfamilies indicated by grey arc. Different colors represent different *Aphidinae* in phylogenetic tree, red palette indicates the tribe *Macrosiphini*, green palette indicates the tribe *Aphidini*. *S*. *graminum*, green; *R*. *padi*, dark sea green; *A*. *glycines*, spring green; *A*. *gossypii*, green yellow; *D*. *noxia*, hot pink; *M*. *persicae*, pink; *S*. *avenae*, dark orange; *A*. *pisum*, red; and *A*. *kondoi*, magenta, *D*. *melanogaster*, blue.
(TIF)

S2 Fig Phylogenetic tree of CCE in *Aphidinae*. CCE is divided into six subfamilies indicated by grey arc. Different colors represent different *Aphidinae* in phylogenetic tree, red palette indicates the tribe *Macrosiphini*, green palette indicates the tribe *Aphidini*. *S*. *graminum*, green; *R*. *padi*, dark sea green; *A*. *glycines*, spring green; *A*. *gossypii*, green yellow; *D*. *noxia*, hot pink; *M*. *persicae*, pink; *S*. *avenae*, dark orange; *A*. *pisum*, red; and *A*. *kondoi*, magenta.
(TIF)

S3 Fig Phylogenetic tree of GST in *Aphidinae*. GST is divided into four subfamilies indicated by grey arc. Different colors represent different *Aphidinae* in phylogenetic tree, red palette indicates the tribe *Macrosiphini*, green palette indicates the tribe *Aphidini*. *S*. *graminum*, green; *R*. *padi*, dark sea green; *A*. *glycines*, spring green; *A*. *gossypii*, green yellow; *D*. *noxia*, hot pink; *M*. *persicae*, pink; *S*. *avenae*, dark orange; *A*. *pisum*, red; and *A*. *kondoi*, magenta.
(TIF)

S4 Fig Phylogenetic tree of P450 in *Aphidinae*. P450 is divided into four clades indicated by the grey arc. Different colors represent different *Aphidinae* in the phylogenetic tree, red palette indicates the tribe *Macrosiphini*, green palette indicates the tribe *Aphidini*. *S*. *graminum*, green; *R*. *padi*, dark sea green; *A*. *glycines*, spring green; *A*. *gossypii*, green-yellow; *D*. *noxia*, hot pink; *M*. *persicae*, pink; *S*. *avenae*, dark orange; *A*. *pisum*, red; and *A*. *kondoi*, magenta.
(TIF)

S5 Fig Phylogenetic tree of UGT in *Aphidinae*. Different colors represent different *Aphidinae* in the phylogenetic tree, red palette indicates the tribe *Macrosiphini*, green palette indicates the tribe *Aphidini*. *S*. *graminum*, green; *R*. *padi*, dark sea green; *A*. *glycines*, spring green; *A*. *gossypii*, green-yellow; *D*. *noxia*, hot pink; *M*. *persicae*, pink; *S*. *avenae*, dark orange; *A*. *pisum*, red; and *A*. *kondoi*, magenta.
(TIF)

S6 Fig Phylogenetic tree of detoxification-related subfamilies in nine *Aphidinae* species. A-D: ABC-A/B/C/G, E-F: P450-CYP3/ mitochondria, G-I: CCE-A/ β-esterase / acetylcholine esterase. Different colors represent different *Aphidinae* in phylogenetic tree, red palette indicates the tribe *Macrosiphini*, green palette indicates the tribe *Aphidini*: *S*. *graminum*, green; *R*. *padi*, dark sea green; *A*. *glycines*, spring green; *A*. *gossypii*, green-yellow; *D*. *noxia*, hot pink; *M*. *persicae*, pink; *S*. *avenae*, dark orange; *A*. *pisum*, red; and *A*. *kondoi*, magenta.
(TIF)

S1 Table Detoxification-related gene numbers of seventeen insect organisms. (XLSX)

## Abbreviations

*A. glycines*: *Aphis glycines*
*A. gossypii*: *Aphis gossypii*
*A. kondoi*: *Acyrthosiphon kondoi*
*A. pisum*: *Acyrthosiphon pisum*
ABC: ATP-binding cassette transporter
*B. tabaci*: *Bemisia tabaci*
CCE: carboxylesterase
*D. noxia*: *Diuraphis noxia*
*D. plexippus*: *Danaus plexippus*
D-site: Docking site
GST: glutathione S-transferase
*L. decemlineata*: *Leptinotarsa decemlineata*
*M. persicae*: *Myzus persicae*
*N. lugens*: *Nilaparvata lugens*
*P. xylostella*: *Plutella xylostella*
P450: cytochrome P450
*R. padi*: *Rhopalosiphum padi*
*S. avenae*: *Sitobion avenae*
*S. graminum*: *Schizaphis graminum*
*S. litura*: *Spodoptera litura*
*T. castaneum*: *Tribolium castaneum*
UGT: UDP-glycosyltransferases

## References

[1] Ramsey JS, Rider DS, Walsh TK, De Vos M, Gordon KH, Ponnala L, et al: Comparative analysis of detoxification enzymes in Acyrthosiphon pisum and Myzus persicae. *Insect Mol Biol* 2010, 19 Suppl 2:155–164. doi: 10.1111/j.1365-2583.2009.00973.x 20482647

[2] Rane RV, Walsh TK, Pearce SL, Jermiin LS, Gordon KH, Richards S, et al: Are feeding preferences and insecticide resistance associated with the size of detoxifying enzyme families in insect herbivores? *Curr Opin Insect Sci* 2016, 13:70–76. doi: 10.1016/j.cois.2015.12.001 27436555

[3] Eakteiman G, Moses-Koch R, Moshitzky P, Mestre-Rincon N, Vassao DG, Luck K, et al: Targeting detoxification genes by phloem-mediated RNAi: A new approach for controlling phloem- feeding insect pests. *Insect Biochem Mol Biol* 2018, 100:10–21. doi: 10.1016/j.ibmb.2018.05.008 29859812

[4] Gaddelapati SC, Kalsi M, Roy A, Palli SR: Cap ’n’ collar C regulates genes responsible for imidacloprid resistance in the Colorado potato beetle, Leptinotarsa decemlineata. *Insect Biochem Mol Biol* 2018, 99:54–62. doi: 10.1016/j.ibmb.2018.05.006 29852222

[5] Ahn SJ, Vogel H, Heckel DG: Comparative analysis of the UDP-glycosyltransferase multigene family in insects. *Insect Biochem Mol Biol* 2012, 42(2):133–147. doi: 10.1016/j.ibmb.2011.11.006 22155036

[6] Pan YO, Guo HL, Gao XW: Carboxylesterase activity, cDNA sequence, and gene expression in malathion susceptible and resistant strains of the cotton aphid, Aphis gossypii. *Comp Biochem Phys B* 2009, 152(3):266–270. 10.1016/j.cbpb.2008.12.002 19110065

[7] Li S, Yu X, Feng Q. Fat Body Biology in the Last Decade. *Annu Rev Entomol*. 2019 Jan 7;64:315–333. doi: 10.1146/annurev-ento-011118-112007 30312553

[8] Urlacher VB, Girhard M: Cytochrome P450 Monooxygenases in Biotechnology and Synthetic Biology. *Trends Biotechnol* 2019. doi: 10.1016/j.tibtech.2019.01.001 30739814

[9] Schuler MA, Berenbaum MR: Structure and function of cytochrome P450S in insect adaptation to natural and synthetic toxins: insights gained from molecular modeling. *J Chem Ecol* 2013, 39(9). 10.1007/s10886-013-0335-7 24036972

[10] Schuler MA: P450s in plant-insect interactions. *Biochim Biophys Acta* 2011, 1814(1):36–45. doi: 10.1016/j.bbapap.2010.09.012 20883828

[11] Pan YO, Chai PJ, Zheng C, Xu HF, Wu YQ, Gao XW, et al: Contribution of cytochrome P450 monooxygenase CYP380C6 to spirotetramat resistance in Aphis gossypii Glover. *Pestic Biochem Phys* 2018, 148:182–189. doi: 10.1016/j.pestbp.2018.04.015 29891371

[12] Wang H, Shi Y, Wang L, Liu S, Wu S, Yang Y, et al: CYP6AE gene cluster knockout in *Helicoverpa armigera* reveals a role in detoxification of phytochemicals and insecticides. *Nat Commun* 2018, 9(1):4820. doi: 10.1038/s41467-018-07226-6 30446639 PMC6240031

[13] Yan LZ, Yang PC, Jiang F, Cui N, Ma EB, Qiao CL, et al: Transcriptomic and phylogenetic analysis of *Culex pipiens quinquefasciatus* for three detoxification gene families. *Bmc Genomics* 2012, 13. doi: 10.1186/1471-2164-13-609 23140097 PMC3505183

[14] Balabanidou V, Kampouraki A, MacLean M, Blomquist GJ, Tittiger C, Juarez MP, et al: Cytochrome P450 associated with insecticide resistance catalyzes cuticular hydrocarbon production in *Anopheles gambiae*. *Proc Natl Acad Sci USA* 2016, 113(33):9268–9273. doi: 10.1073/pnas.1608295113 27439866 PMC4995928

[15] Xu LJ, Duan XL, Lv YH, Zhang XH, Nie ZS, Xie CJ, et al: Silencing of an aphid carboxylesterase gene by use of plant-mediated RNAi impairs Sitobion avenae tolerance of Phoxim insecticides. *Transgenic Res* 2014, 23(2):389–396. doi: 10.1007/s11248-013-9765-9 24242160

[16] Feng X, Li M, Liu N: Carboxylesterase genes in pyrethroid resistant house flies, Musca domestica. *Insect Biochem Mol Biol* 2018, 92:30–39. doi: 10.1016/j.ibmb.2017.11.007 29154832

[17] Gong YH, Ai GM, Li M, Shi XY, Diao QY, Gao XW: Functional characterization of carboxylesterase gene mutations involved in Aphis gossypii resistance to organophosphate insecticides. *Insect Mol Biol* 2017, 26(6):702–714. doi: 10.1111/imb.12331 28799241

[18] Shi HX, Pei LH, Gu SS, Zhu SC, Wang YY, Zhang Y, Li B: Glutathione S-transferase (GST) genes in the red flour beetle, Tribolium castaneum, and comparative analysis with five additional insects. *Genomics* 2012, 100(5):327–335. doi: 10.1016/j.ygeno.2012.07.010 22824654

[19] Nicholson SJ, Nickerson ML, Dean M, Song Y, Hoyt PR, Rhee H, et al: The genome of *Diuraphis noxia*, a global aphid pest of small grains. *Bmc Genomics* 2015, 16:429. doi: 10.1186/s12864-015-1525-1 26044338 PMC4561433

[20] Arockiaraj J, Gnanam AJ, Palanisamy R, Bhatt P, Kumaresan V, Chaurasia MK, et al: A cytosolic glutathione s-transferase, GST-theta from freshwater prawn *Macrobrachium rosenbergii*: molecular and biochemical properties. *Gene* 2014, 546(2):437–442. doi: 10.1016/j.gene.2014.05.063 24879918

[21] Bock KW: The UDP-glycosyltransferase (UGT) superfamily expressed in humans, insects and plants: Animal-plant arms-race and co-evolution. *Biochem Pharmacol* 2016, 99:11–17. doi: 10.1016/j.bcp.2015.10.001 26453144

[22] Meech R, Hu DG, McKinnon RA, Mubarokah SN, Haines AZ, Nair PC, et al: The UDP- Glycosyltransferase (UGT) Superfamily: New Members, New Functions, and Novel Paradigms. *Physiol Rev* 2019, 99(2):1153–1222. doi: 10.1152/physrev.00058.2017 30724669

[23] Huang FF, Chai CL, Zhang Z, Liu ZH, Dai FY, Lu C, et al: The UDP-glucosyltransferase multigene family in Bombyx mori. *Bmc Genomics* 2008, 9:563. doi: 10.1186/1471-2164-9-563 19038024 PMC2633020

[24] Cheng T, Wu J, Wu Y, Chilukuri RV, Huang L, Yamamoto K, et al: Genomic adaptation to polyphagy and insecticides in a major East Asian noctuid pest. *Nat Ecol Evol* 2017, 1(11):1747–1756. doi: 10.1038/s41559-017-0314-4 28963452

[25] Dermauw W, Van Leeuwen T: The ABC gene family in arthropods: comparative genomics and role in insecticide transport and resistance. *Insect Biochem Mol Biol* 2014, 45:89–110. doi: 10.1016/j.ibmb.2013.11.001 24291285

[26] Tian L, Song T, He R, Zeng Y, Xie W, Wu Q, et al: Genome-wide analysis of ATPbinding cassette (ABC) transporters in the sweetpotato whitefly, *Bemisia tabaci*. *Bmc Genomics* 2017, 18(1):330. doi: 10.1186/s12864-017-3706-6 28446145 PMC5405539

[27] Liu S, Zhou S, Tian L, Guo E, Luan Y, Zhang J, et al: Genome-wide identification and characterization of ATP- binding cassette transporters in the silkworm, *Bombyx mori*. *Bmc Genomics* 2011, 12:491. doi: 10.1186/1471-2164-12-491 21981826 PMC3224256

[28] Qi W, Ma X, He W, Chen W, Zou M, Gurr GM, et al: Characterization and expression profiling of ATP-binding cassette transporter genes in the diamondback moth, *Plutella xylostella* (L.). *Bmc Genomics* 2016, 17(1):760. doi: 10.1186/s12864-016-3096-1 27678067 PMC5039799

[29] Quan Q, Hu X, Pan B, Zeng B, Wu N, Fang G, et al: Draft genome of the cotton aphid *Aphis gossypii*. *Insect Biochem Mol Biol* 2019, 105:25–32. doi: 10.1016/j.ibmb.2018.12.007 30590189

[30] Liu XD, Xu TT, Lei HX: Refuges and host shift pathways of host-specialized aphids *Aphis gossypii*. *Sci Rep* 2017, 7(1):2008. doi: 10.1038/s41598-017-02248-4 28515483 PMC5435715

[31] Jones P, Binns D, Chang HY, Fraser M, Li WZ, McAnulla C, et al: InterProScan 5: genome-scale protein function classification. *Bioinformatics* 2014, 30(9): doi: 10.1093/bioinformatics/btu031 . 24451626 PMC3998142

[32] Li S, Zhu S, Jia Q, Yuan D, Ren C, Li K, et al: The genomic and functional landscapes of developmental plasticity in the American cockroach. *Nat Commun* 2018, 9(1):1008. doi: 10.1038/s41467-018-03281-1 29559629 PMC5861062

[33] von Dohlen Carol D., Rowe Carol A., Heie Ole E.: A test of morphological hypotheses for tribal and subtribal relationships of Aphidinae (Insecta: Hemiptera: *Aphididae*) using DNA sequences. *Molecular Phylogenetics and Evolution* 2006,38:316–329. doi: 10.1016/j.ympev.2005.04.035 16368250

[34] Behura SK: Insect phylogenomics. *Insect Mol Biol*. 2015 Aug;24(4):403–11. doi: 10.1111/imb.12174 25963452 PMC4503476

[35] Rispe C, Legeai F, Nabity PD, Fernández R, Arora AK, Baa-Puyoulet P, et al: The genome sequence of the grape phylloxera provides insights into the evolution, adaptation, and invasion routes of an iconic pest. *BMC Biol*. 2020 Jul 23;18(1):90. doi: 10.1186/s12915-020-00820-5 32698880 PMC7376646

[36] Xie W, Chen C, Yang Z, Guo L, Yang X, Wang D, et al: Genome sequencing of the sweetpotato whitefly *Bemisia tabaci* MED/Q. *Gigascience* 2017, 6(5):1–7. doi: 10.1093/gigascience/gix018 28327996 PMC5467035

[37] Ye YX, Zhang HH, Li DT, Zhuo JC, Shen Y, Hu QL, et al: Chromosome-level assembly of the brown planthopper genome with a characterized Y chromosome. *Mol Ecol Resour*. 2021 May;21(4):1287–1298. doi: 10.1111/1755-0998.13328 33460519

[38] Tribolium Genome Sequencing Consortium, Richards S, Gibbs RA, Weinstock GM, et al. The genome of the model beetle and pest *Tribolium castaneum*. *Nature*. 2008 Apr 24;452(7190):949–55. doi: 10.1038/nature06784 18362917

[39] Schoville SD, Chen YH, Andersson MN, Benoit JB, Bhandari A, Bowsher JH, et al: A model species for agricultural pest genomics: the genome of the Colorado potato beetle, *Leptinotarsa decemlineata* (Coleoptera: Chrysomelidae). *Sci Rep*. 2018 Jan 31;8(1):1931. doi: 10.1038/s41598-018-20154-1 29386578 PMC5792627

[40] Ward CM, Perry KD, Baker G, Powis K, Heckel DG, Baxter SW: A haploid diamondback moth (*Plutella xylostella* L.) genome assembly resolves 31 chromosomes and identifies a diamide resistance mutation. *Insect Biochem Mol Biol*. 2021 Nov;138:103622. doi: 10.1016/j.ibmb.2021.103622 34252570

[41] Zhan S, Zhang W, Niitepõld K, Hsu J, Haeger JF, Zalucki MP: The genetics of monarch butterfly migration and warning coloration. *Nature*.2014 Oct 16;514(7522):317–21. doi: 10.1038/nature13812 25274300 PMC4331202

[42] Chen K, Durand D, Farach-Colton M: NOTUNG: a program for dating gene duplications and optimizing gene family trees. J Comput Biol 2000, 7(3–4):429–447. doi: 10.1089/106652700750050871 11108472

[43] Mathers TC, Chen Y, Kaithakottil G, Legeai F, Mugford ST, Baa-Puyoulet P, et al: Rapid transcriptional plasticity of duplicated gene clusters enables a clonally reproducing aphid to colonize diverse plant species. *Genome Biol* 2017,18(1):27. doi: 10.1186/s13059-016-1145-3 28190401 PMC5304397

[44] Duan X, Wang K, Su S, Tian R, Li Y, Chen M: De novo transcriptome analysis and microsatellite marker development for population genetic study of a serious insect pest, *Rhopalosiphum padi* (L.) (Hemiptera: *Aphididae*). *Plos One* 2017, 12(2): e0172513. doi: 10.1371/journal.pone.0172513 28212394 PMC5315398

[45] Wenger Jacob A, Cassone Bryan J, Legeai Fabrice, Johnston J Spencer, Bansal Raman, Yates Ashley D, et al: Whole genome sequence of the soybean aphid, *Aphis glycines*. *Insect Biochem Mol Biol*. 2020,123:102917. doi: 10.1016/j.ibmb.2017.01.005 28119199

[46] Godfray HC: The pea aphid genome. *Insect Mol Biol* 2010, 19 Suppl 2:1–4. doi: 10.1111/j.1365-2583.2009.00980.x 20482634

[47] Peccoud J, Simon JC, McLaughlin HJ, Moran NA. Post-Pleistocene radiation of the pea aphid complex revealed by rapidly evolving endosymbionts. *Proc Natl Acad Sci U S A*. 2009 Sep 22;106(38):16315–20. doi: 10.1073/pnas.0905129106 19805299 PMC2752580

[48] Guo SM, Kamphuis LG, Gao LL, Klingler JP, Lichtenzveig J, Edwards O, et al: Identification of distinct quantitative trait loci associated with defence against the closely related aphids *Acyrthosiphon pisum* and *A*. *kondoi* in *Medicago truncatula*. *J Exp Bot* 2012, 63(10):3913–3922. doi: 10.1093/jxb/ers084 22442407 PMC3388833

[49] Zhang L, Lu H, Guo K, Yao S, Cui F: Insecticide resistance status and detoxification enzymes of wheat aphids *Sitobion avenae* and *Rhopalosiphum padi*. *Sci China Life Sci* 2017, 60(8):927–930. doi: 10.1007/s11427-017-9105-x 28755297

[50] Zhang Y, Fu Y, Wang Q, Liu X, Li Q, Chen J: Transcriptome analysis reveals rapid defence responses in wheat induced by phytotoxic aphid *Schizaphis graminum* feeding. *Bmc Genomics* 2020, 21(1):339. doi: 10.1186/s12864-020-6743-5 32366323 PMC7199342

[51] Yates AD, Michel A: Mechanisms of aphid adaptation to host plant resistance. *Curr Opin Insect Sci* 2018, 26:41–49. doi: 10.1016/j.cois.2018.01.003 29764659

[52] Zhu F, Moural TW, Shah K, Palli SR: Integrated analysis of cytochrome P450 gene superfamily in the red flour beetle, *Tribolium castaneum*. *Bmc Genomics* 2013, 14:174. doi: 10.1186/1471-2164-14-174 23497158 PMC3682917

[53] Zhou X, Slone JD, Rokas A, Berger SL, Liebig J, Ray A, et al: Phylogenetic and transcriptomic analysis of chemosensory receptors in a pair of divergent ant species reveals sex specific signatures of odor coding. *PLoS Genet* 2012, 8(8):e1002930. doi: 10.1371/journal.pgen.1002930 22952454 PMC3431598

[54] Francis F, Haubruge E, Gaspar C, Dierickx PJ: Glutathione S-transferases of *Aulacorthum solani* and *Acyrthosiphon pisum*: partial purification and characterization. *Comp Biochem Physiol B Biochem Mol Biol* 2001, 129(1):165–171. doi: 10.1016/s1096-4959(01)00329-3 11337260

[55] Lee SY, Lim IA, Kang GU, Cha SJ, Altanbyek V, Kim HJ, et al: Protective effect of Drosophila glutathione transferase omega 1 against hydrogen peroxide-induced neuronal toxicity. *Gene* 2015, 568(2):203–210. doi: 10.1016/j.gene.2015.05.058 26024591

[56] Yan HR, Meng F, Jia HH, Guo XQ, Xu BH: The identification and oxidative stress response of a zeta class glutathione S-transferase (GSTZ1) gene from *Apis cerana cerana*. *J Insect Physiol* 2012, 58(6):782–791. doi: 10.1016/j.jinsphys.2012.02.003 22360998

[57] Wang YJ, Qiu L, Ranson H, Lumjuan N, Hemingway J, Setzer WN, et al: Structure of an insect epsilon class glutathione S-transferase from the malaria vector *Anopheles gambiae* provides an explanation for the high DDT-detoxifying activity. *J Struct Biol* 2008, 164(2):228–235. doi: 10.1016/j.jsb.2008.08.003 18778777

[58] Yan SG, Cui F, Qiao CL: Structure, Function and Applications of Carboxylesterases from Insects for Insecticide Resistance. *Protein and Peptide Letters* 2009, 16(10):1181–1188. doi: 10.2174/092986609789071243 19508184

[59] Oakeshott JG, Johnson RM, Berenbaum MR, Ranson H, Cristino AS, Claudianos C: Metabolic enzymes associated with xenobiotic and chemosensory responses in *Nasonia vitripennis*. *Insect Molecular Biology* 2010, 19:147–163. doi: 10.1111/j.1365-2583.2009.00961.x 20167025

[60] Chen CY, Wang CC, Liu Y, Shi XY, Gao XW: Transcriptome analysis and identification of P450 genes relevant to imidacloprid detoxification in *Bradysia odoriphaga*. *Sci Rep-Uk* 2018, 8. doi: 10.1038/s41598-018-20981-2 29416091 PMC5803201

