# Supplementary figures and images for: The phylogenetic and evolutionary analyses of detoxification gene families in Aphidinae species
Source: PLoS One. 2022 Feb 10;17(2):e0263462. doi: 10.1371/journal.pone.0263462 (PMC8830634; doi:10.1371/journal.pone.0263462)

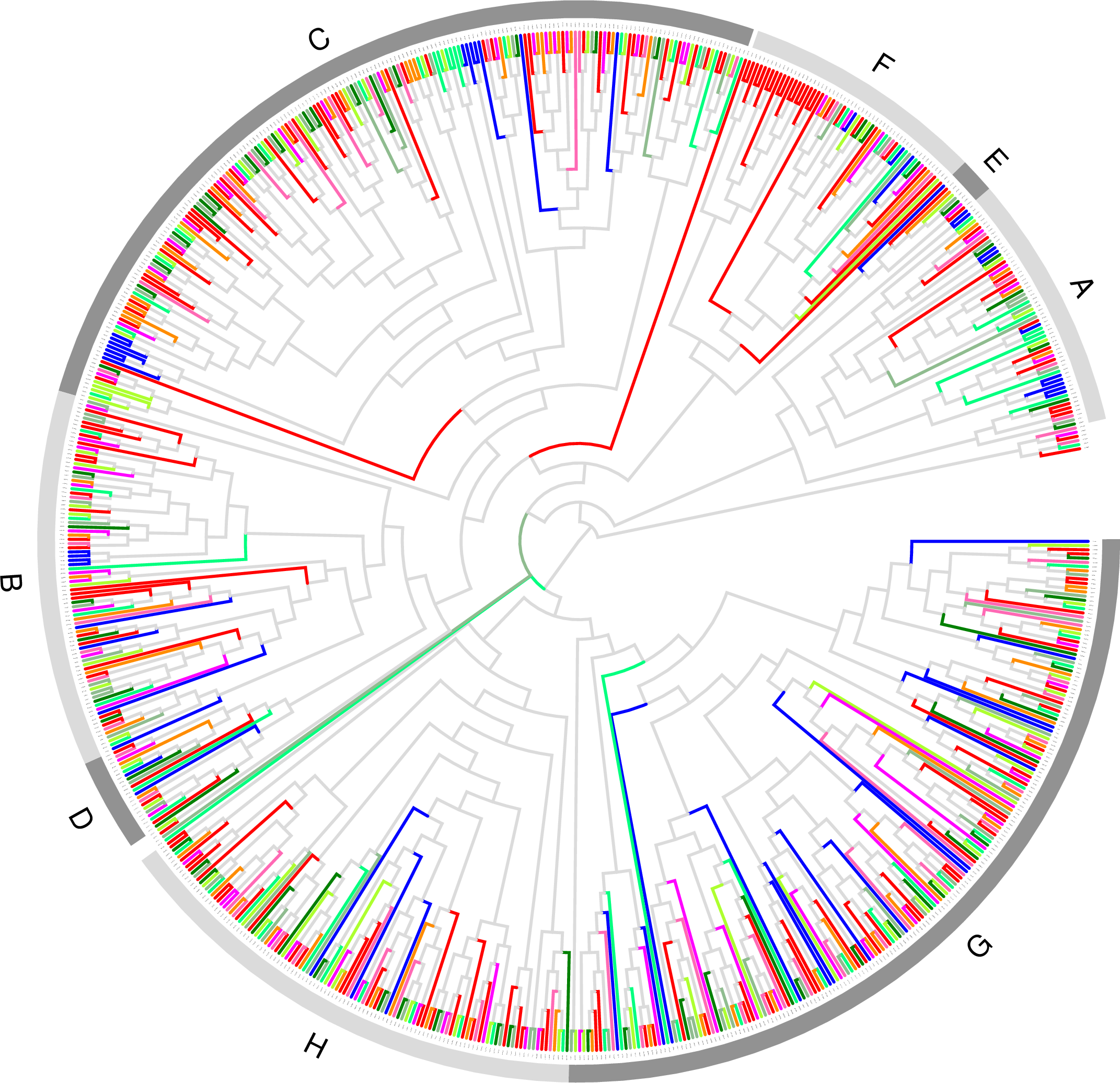

Supplement: S1 Fig — ABC is divided into eight subfamilies indicated by grey arc. Different colors represent different Aphidinae in phylogenetic tree, red palette indicates the tribe Macrosiphini, green palette indicates the tribe Aphidini. S. graminum, green; R. padi, dark sea green; A. glycines, spring green; A. gossypii, green yellow; D. noxia, hot pink; M. persicae, pink; S. avenae, dark orange; A. pisum, red; and A. kondoi, magenta, D. melanogaster, blue. (TIF) [file pone.0263462.s001.tif]

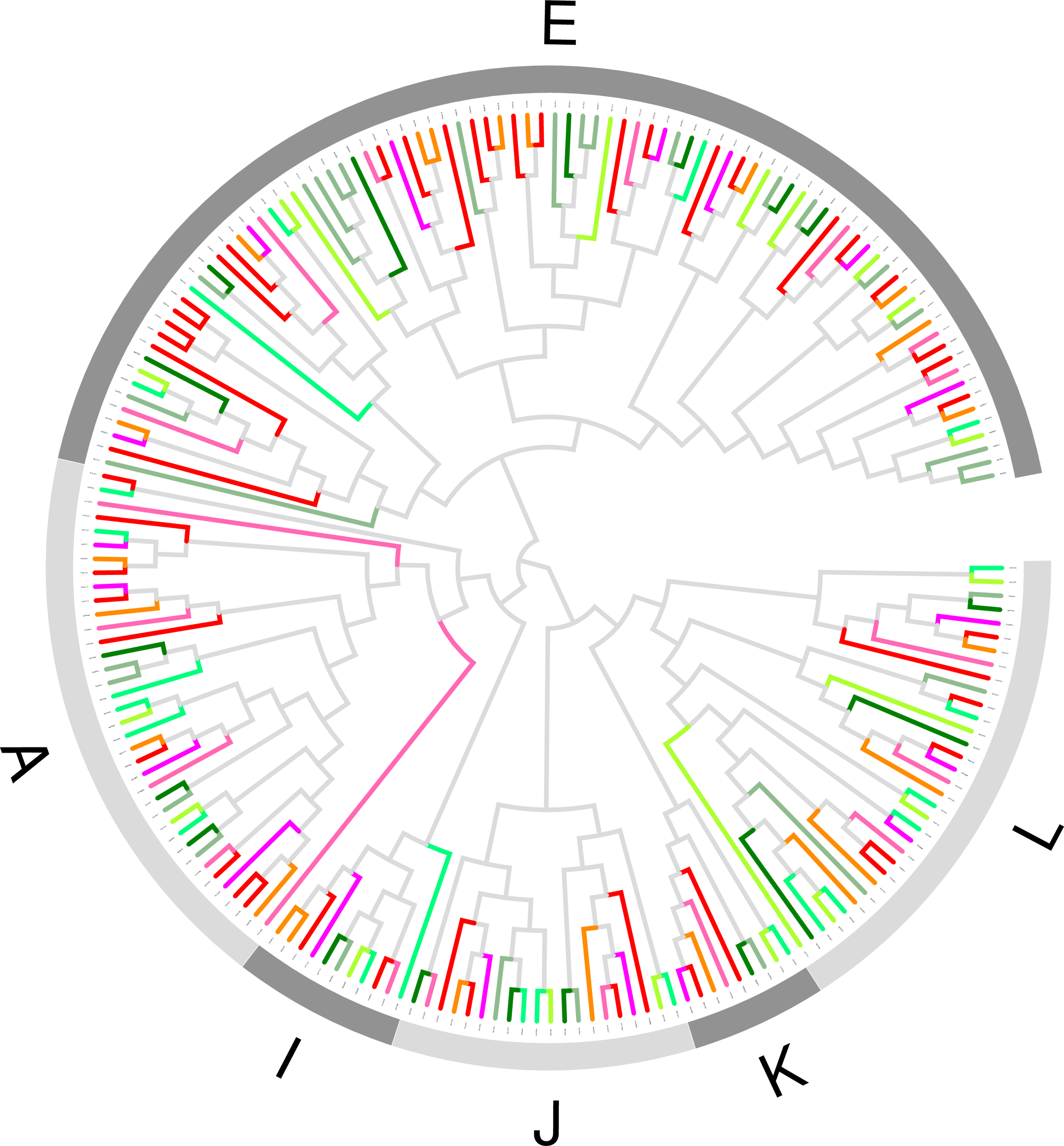

Supplement: S2 Fig — CCE is divided into six subfamilies indicated by grey arc. Different colors represent different Aphidinae in phylogenetic tree, red palette indicates the tribe Macrosiphini, green palette indicates the tribe Aphidini. S. graminum, green; R. padi, dark sea green; A. glycines, spring green; A. gossypii, green yellow; D. noxia, hot pink; M. persicae, pink; S. avenae, dark orange; A. pisum, red; and A. kondoi, magenta. (TIF) [file pone.0263462.s002.tif]

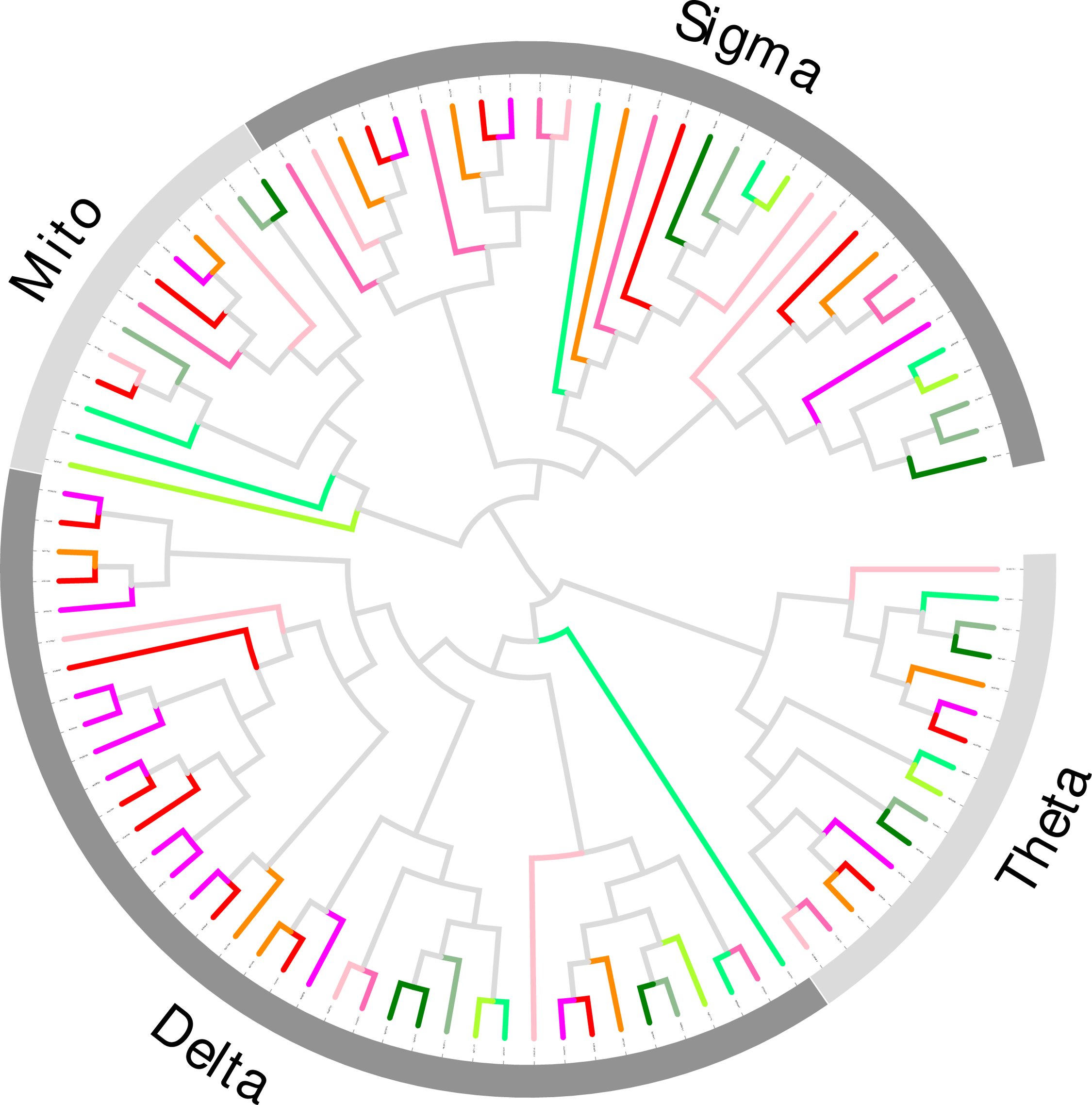

Supplement: S3 Fig — GST is divided into four subfamilies indicated by grey arc. Different colors represent different Aphidinae in phylogenetic tree, red palette indicates the tribe Macrosiphini, green palette indicates the tribe Aphidini. S. graminum, green; R. padi, dark sea green; A. glycines, spring green; A. gossypii, green yellow; D. noxia, hot pink; M. persicae, pink; S. avenae, dark orange; A. pisum, red; and A. kondoi, magenta. (TIF) [file pone.0263462.s003.tif]

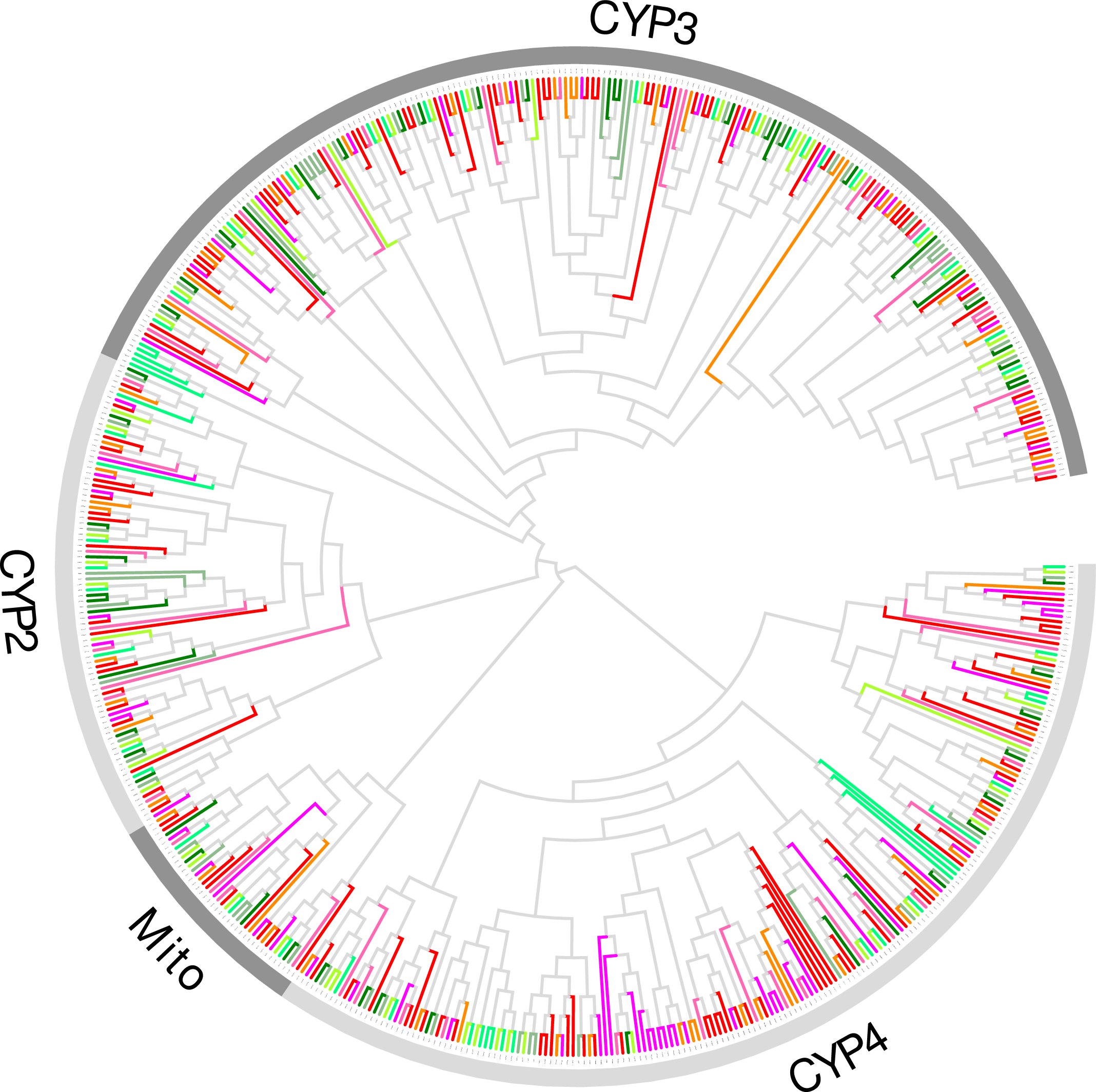

Supplement: S4 Fig — P450 is divided into four clades indicated by the grey arc. Different colors represent different Aphidinae in the phylogenetic tree, red palette indicates the tribe Macrosiphini, green palette indicates the tribe Aphidini. S. graminum, green; R. padi, dark sea green; A. glycines, spring green; A. gossypii, green-yellow; D. noxia, hot pink; M. persicae, pink; S. avenae, dark orange; A. pisum, red; and A. kondoi, magenta. (TIF) [file pone.0263462.s004.tif]

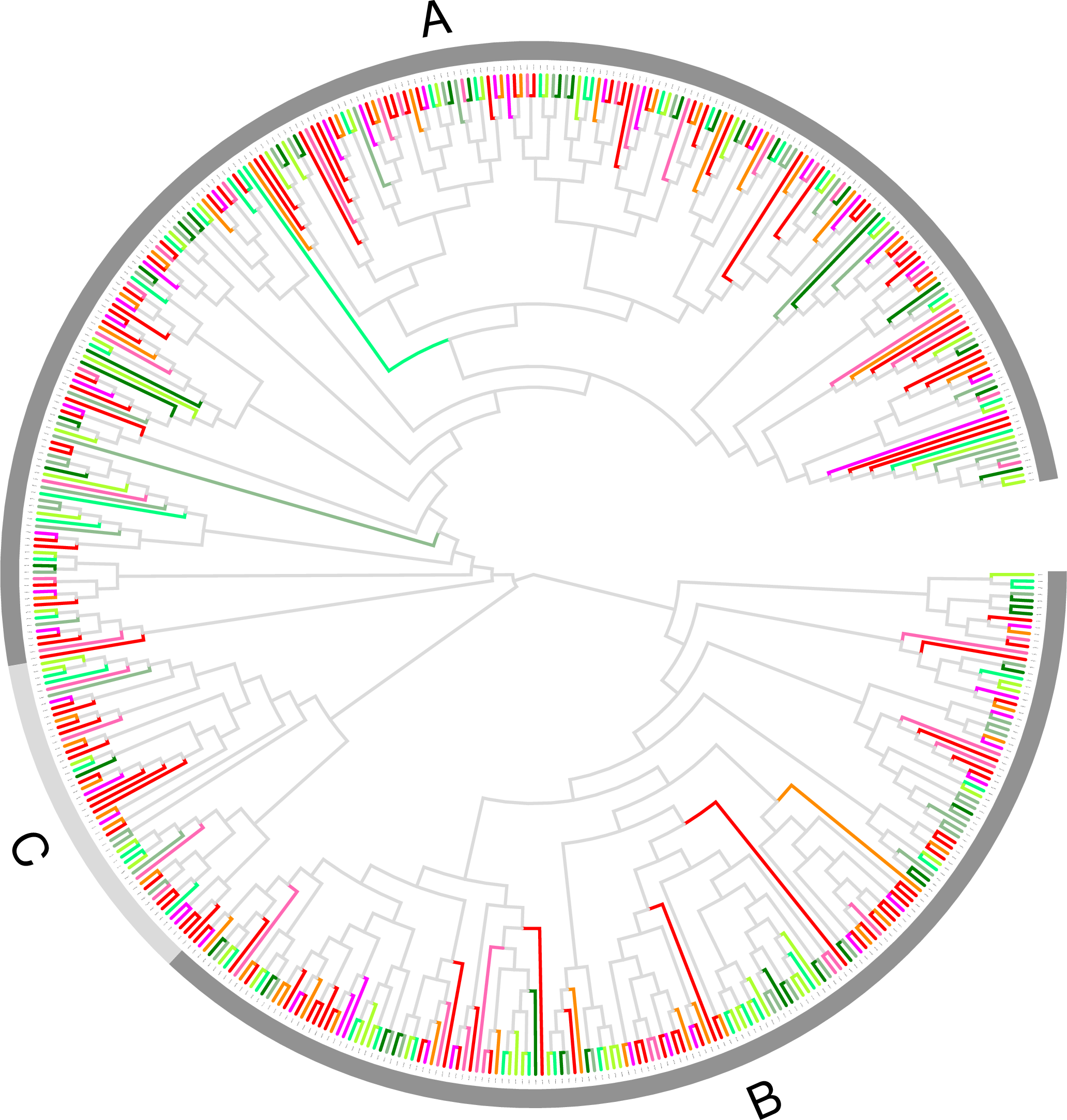

Supplement: S5 Fig — Different colors represent different Aphidinae in the phylogenetic tree, red palette indicates the tribe Macrosiphini, green palette indicates the tribe Aphidini. S. graminum, green; R. padi, dark sea green; A. glycines, spring green; A. gossypii, green-yellow; D. noxia, hot pink; M. persicae, pink; S. avenae, dark orange; A. pisum, red; and A. kondoi, magenta. (TIF) [file pone.0263462.s005.tif]

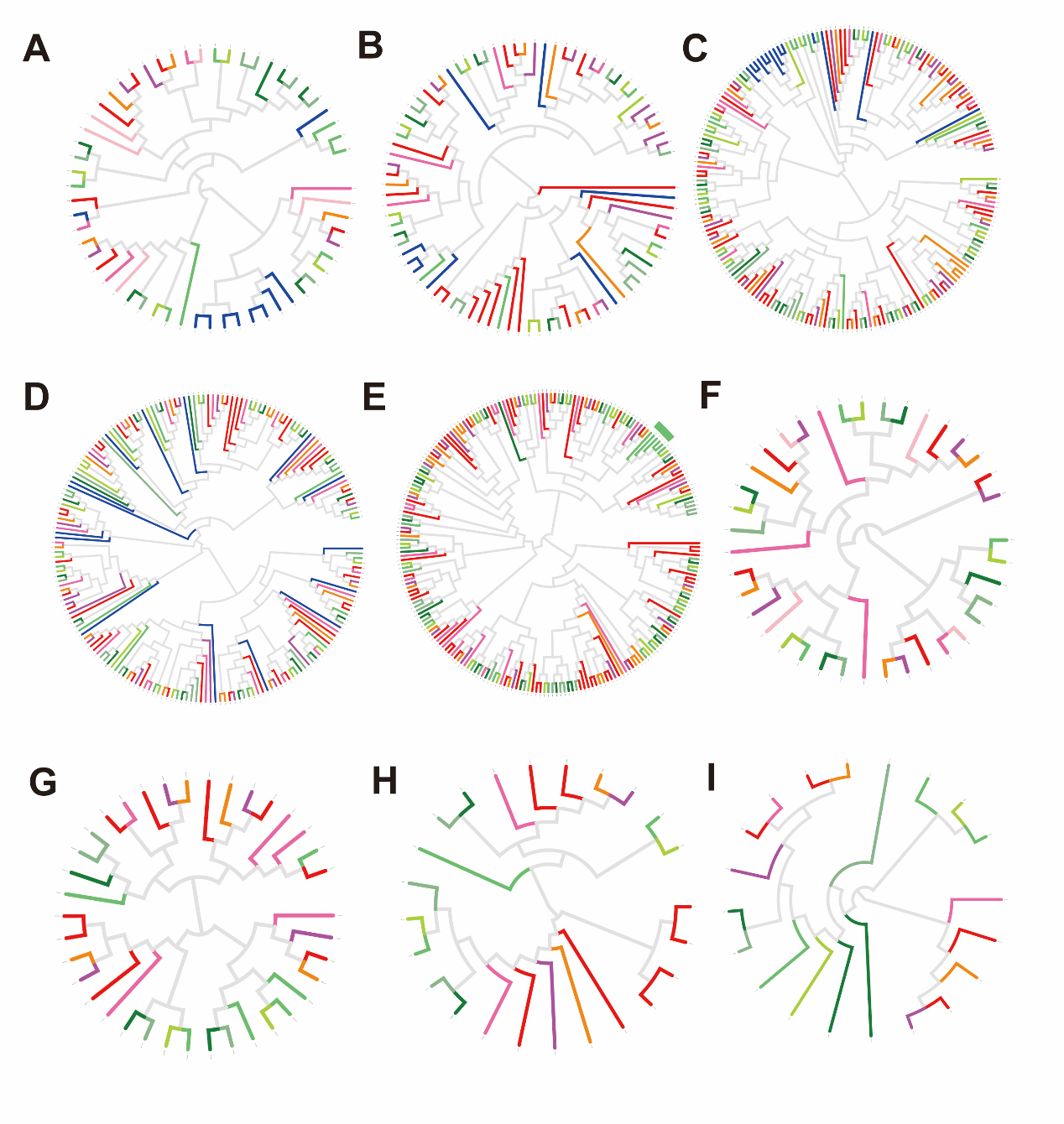

Supplement: S6 Fig — A-D: ABC-A/B/C/G, E-F: P450-CYP3/ mitochondria, G-I: CCE-A/ β-esterase / acetylcholine esterase. Different colors represent different Aphidinae in phylogenetic tree, red palette indicates the tribe Macrosiphini, green palette indicates the tribe Aphidini: S. graminum, green; R. padi, dark sea green; A. glycines, spring green; A. gossypii, green-yellow; D. noxia, hot pink; M. persicae, pink; S. avenae, dark orange; A. pisum, red; and A. kondoi, magenta. (TIF) [file pone.0263462.s006.tif]
